# Application of adaptive design and decision making to a phase II trial of a phosphodiesterase inhibitor for the treatment of intermittent claudication

**DOI:** 10.1186/1745-6215-12-134

**Published:** 2011-05-25

**Authors:** Roger J Lewis, Jason T Connor, John R Teerlink, James R Murphy, Leslie T Cooper, William R Hiatt, Eric P Brass

**Affiliations:** 1 Department of Emergency Medicine, Harbor-UCLA Medical Center, 1000 West Carson Street, Torrance, California 90509 USA; 2 Department of Medicine, David Geffen School of Medicine at UCLA, 10833 Le Conte Avenue, Los Angeles, California 90095 USA; 3 Los Angeles Biomedical Research Institute, 1124 West Carson Street, Torrance, California 90502 USA; 4 Berry Consultants, LLC, 9757 Cypress Pine Street, Orlando, Florida 32827 USA; 5 College of Medicine, University of Central Florida, 6850 Lake Nona Boulevard, Orlando, Florida 32827 USA; 6 Section of Cardiology, San Francisco Veterans Affairs Medical Center, 4150 Clement Street, San Francisco, California 94121 USA; 7 Department of Medicine, School of Medicine, University of California San Francisco, 505 Parnassus Avenue, San Francisco, California 94143 USA; 8 Division of Biostatistics and Bioinformatics, National Jewish Medical and Research Center, 1400 Jackson Street, Denver, Colorado 80206 USA; 9 Gonda Vascular Center and Division of Cardiovascular Diseases, Mayo Clinic, 200 First Street SW, Rochester, MN 55906, USA; 10 Division of Cardiology, Department of Medicine, University of Colorado School of Medicine, 12631 East 17th Avenue, Aurora, Colorado, 80045, USA; 11 CPC Clinical Research, 13199 East Montview Boulevard, Suite 200, Aurora, Colorado 80045 USA; 12 Department of Medicine, Harbor-UCLA Medical Center, 1000 West Carson Street, Torrance, California 90502 USA

## Abstract

**Background:**

Claudication secondary to peripheral artery disease (PAD) is associated with substantial functional impairment. Phosphodiesterase (PDE) inhibitors have been shown to increase walking performance in these patients. K-134 is a selective PDE 3 inhibitor being developed as a potential treatment for claudication. The use of K-134, as with other PDE 3 inhibitors, in patients with PAD raises important safety and tolerability concerns, including the induction of cardiac ischemia, tachycardia, and hypotension. We describe the design, oversight, and implementation of an adaptive, phase II, dose-finding trial evaluating K-134 for the treatment of stable, intermittent claudication.

**Methods:**

The study design was a double-blind, multi-dose (25 mg, 50 mg, and 100 mg of K-134), randomized trial with both placebo and active comparator arms conducted in the United States and Russia. The primary objective of the study was to compare the highest tolerable dose of K-134 versus placebo using peak walking time after 26 weeks of therapy as the primary outcome. Study visits with intensive safety assessments were included early in the study period to provide data for adaptive decision making. The trial used an adaptive, dose-finding strategy to efficiently identify the highest dose(s) most likely to be safe and well tolerated, based on the side effect profiles observed within the trial, so that less promising doses could be abandoned. Protocol specified criteria for safety and tolerability endpoints were used and modeled prior to the adaptive decision making. The maximum target sample size was 85 subjects in each of the retained treatment arms.

**Results:**

When 199 subjects had been randomized and 28-day data were available from 143, the Data Monitoring Committee (DMC) recommended termination of the lowest dose (25 mg) treatment arm. Safety evaluations performed during 14- and 28-day visits which included in-clinic dosing and assessments at peak drug concentrations provided core data for the DMC review. At the time of review, no subject in any of the five treatment arms (placebo, three K-134-containing arms, and cilostazol) had met pre-specified definitions for resting tachycardia or ischemic changes on exercise ECG. If, instead of dropping the 25-mg K-134 treatment arm, all arms had been continued to full enrollment, then approximately 43 additional research subjects would have been required to complete the trial.

**Conclusions:**

In this phase II, dose-finding trial of K-134 in the treatment of stable intermittent claudication, no concerning safety signals were seen at interim analysis, allowing the discontinuation of the lowest-dose-containing arm and the retention of the two highest-dose-containing arms. The adaptive design facilitated safe and efficient evaluation of K-134 in this high-risk cardiovascular population.

**Trial registration:**

ClinicalTrials.gov: NCT00783081

## Introduction

Peripheral artery disease (PAD) is a common manifestation of systemic atherosclerotic disease, and is associated with both coronary and carotid arterial disease leading to increased risk of myocardial infarction, stroke and death [1,2]. Medical treatment of PAD includes management of cardiovascular risk factors and the use of antiplatelet agents to reduce the risk of myocardial infarction and ischemic stroke.

Approximately one-third of patients with PAD suffer from claudication, typified by pain in one or both legs that is brought on by walking and relieved by rest [3]. Claudication is associated with decreased functional capacity, impairment of activities of daily living, and reduced quality of life. Currently, cilostazol is the only guideline-recommended pharmacologic agent approved in the United States for the treatment of claudication [4]. Cilostazol is a phosphodiesterase (PDE) 3 inhibitor with vasodilatory and antiplatelet activity. Treatment with cilostazol is associated with both an increase in peak treadmill performance and an improvement in quality of life [5]. Another PDE 3 inhibitor, NM-702, has been evaluated in a phase II study with positive results [6]. However, treatment with PDE 3 inhibitors can cause adverse effects; cilostazol can cause orthostatic hypotension, tachycardia, palpitations and headache. In patients with underlying vascular disease, the induction of hypotension and tachycardia raise concerns for induction of ischemic events. Perhaps related to this, the PDE 3 inhibitor milrinone has been associated with increased mortality in patients with severe heart failure [7].

K-134 is a selective PDE 3 inhibitor that in Phase I trials involving healthy volunteers has the expected vasodilatory effects and appears to have more pronounced antiplatelet effects than cilostazol. Although PDE 3 inhibitors have been used successfully to treat claudication, this class of agents raises important safety concerns when used in a population at high risk for underlying cardiovascular disease. Thus, a clinical trial supporting early drug development of a PDE 3 inhibitor, especially in patients suffering from claudication, must be designed to assess potential safety concerns while minimizing risks to study participants. The risk-benefit analysis is made more difficult, however, by the fact that some adverse effects (e.g., hemodynamic changes) are likely to occur quite early in treatment, while the beneficial effect may require six months or longer to fully develop. Additionally, safety and tolerability data from healthy subjects may not be predictive of effects in patients with claudication.

We describe the novel design, oversight, and adaptive decision process associated with a phase II, dose-finding trial evaluating K-134 for the treatment of stable, intermittent claudication. Because of limited prior clinical experience with K-134 in the target population, the ethical imperative to rapidly eliminate from further use any treatments or dosing strategies that are unsafe or poorly tolerated, and the limited availability of patients with stable intermittent claudication for recruitment, an adaptive approach was used to rapidly identify and restrict randomization to the maximum safe and well-tolerated doses of K-134 in the target population [8]. The purpose of this article is to illustrate the capability of a customized adaptive clinical trial design to use active and ongoing assessment of safety and tolerability to better inform interim decision making regarding the termination of both specific dosing strategies and, potentially, the clinical trial itself [8,9]. The primary results of the clinical trial will be published separately.

## Methods

### Study Design and Setting

The study design was a parallel treatment, multi-dose, randomized trial with both placebo and active comparator arms. A Steering Committee oversaw the design, conduct of the trial, and data analysis. By Charter the trial's Data Monitoring Committee reported to the Steering Committee. Kowa Research Institute (KRI) maintained overall regulatory responsibility for the study and contracted with an academic research organization to implement the trial. The trial incorporated the planned, adaptive elimination of one or more treatment arms based on predefined safety and tolerability endpoints. The adaptive aspects of the trial design were developed collaboratively by the trial's Steering Committee, Kowa Research Institute, and the Data Monitoring Committee. The voting statistician member of the Data Monitoring Committee conducted simulations of the adaptive design and performed the required dose-response modeling during conduct of the trial. The study was conducted in 25 sites in the United States and 23 sites in Russia.

### Patient Population

The eligible patient population included patients ages 40 years or greater with a diagnosis of intermittent claudication confirmed by both history and one of the following: (1) a resting ankle brachial index (ABI) ≤ 0.90 at screening; (2) a resting ABI ≥ 0.90 and ≤ 1.00 with a reduction of ≥ 0.20 when measured one minute after treadmill exercise; or (3) an ankle brachial index > 0.90 and a resting toe brachial index < 0.70 in diabetic patients. The symptoms of claudication were required to be stable for three months prior to screening and the subject must have demonstrated a highest peak walking time between one and twelve minutes. Subjects were excluded if they exhibited signs or symptoms of critical leg ischemia, had undergone recent revascularization procedures or coronary artery bypass grafting, or had unstable cardiovascular disease, a history of congestive heart failure or the presence of congestive heart failure as defined by the modified Framingham criteria. Additional exclusion criteria were defined to avoid enrolling patients at additional risk from participation or in whom claudication-limited treadmill performance could not be ascertained.

In the United States, the trial was approved by a central Institutional Review Board (IRB), Western IRB (WIRB) in Olympia, Washington, and by individual local IRBs according to the requirements of each site. In Russia, the trial was approved by the Russian National Ethics Committee and institutional ethics committees (IECs) as required by each site. Voluntary, written informed consent was obtained from each research subject prior to performing any study-related procedures.

### Treatment Arms

Patients were randomized to one of five treatment arms: (1) placebo; (2) K-134 25 mg twice daily (referred to as the 25 mg arm); (3) K-134 25 mg twice daily with a forced titration to 50 mg twice daily after two weeks (referred to as the 50 mg arm); (4) K-134 50 mg twice daily with a forced titration to 100 mg twice daily (referred to as the 100 mg arm); or (5) cilostazol 100 mg twice daily. All drugs were administered orally. The purpose of the forced titration dosing strategy, used in the two higher-dose K-134 arms, was to allow subjects time to acclimate to the pharmacologic effects of K-134 and maximize tolerability. This strategy allowed a safety assessment at the day-14 visit, prior to up-titration. Patients were randomized, after the successful completion of prerequisite baseline evaluation, using an interactive voice recognition system (IVRS) as is typical for large, multicenter, international clinical trials.

### Trial Objectives

The primary objective of the study was to compare the highest safe and tolerable dose of K-134 versus placebo using peak walking time after 26 weeks of therapy as the primary endpoint. The primary outcome was measured as a change from baseline in peak walking time, utilizing a graded exercise treadmill test with a modified Gardner protocol. Secondary objectives included evaluating the safety and efficacy of different doses of K-134 versus placebo at 26 weeks, comparing the safety and efficacy of K-134 versus cilostazol, and exploring the pharmacodynamics of K-134.

### Safety and Tolerability Endpoints for Adaptive Decision Making

The trial used an adaptive, dose-finding strategy to efficiently identify the dose(s) most likely to be safe and well tolerated, based on the side effect profiles observed within the trial, so that less promising doses could be rapidly abandoned and study resources could be utilized most efficiently. The target sample size for each retained study arm was 85 subjects, with the final sample size depending on the number of retained arms.

The target sample size of 85 in each of the study arms was determined based on prospective power calculations under a variety of projected effect sizes, assuming a single pairwise comparison of the highest retained dose of K-134 to placebo (80% power if K-134 increased peak walking time by 40% and 42% power if peak walking time increased by 30%, assuming 20% discontinuation rate). Additionally, the number was felt to be adequate to ensure adequate information on the dose ranging of K-134.

The goal of the adaptive design was to drop from the trial, as quickly as possible, any K-134 dosing regimens that had unacceptable rates of adverse effects or were poorly tolerated. If all arms were safe and well tolerated then the lowest dose was to be dropped. Given the absence of previous experience with K-134 in the PAD population and the known pharmacodynamics of the class, the protocol incorporated safety check points at day 14 (prior to the dose increase in the 50 mg and 100 mg K-134 arms), and at day 28 after two weeks of treatment with the assigned dose. Although, atypical for claudication studies, the 28 day visit included a peak treadmill test conducted at peak drug concentrations with ECG monitoring to allow a comprehensive safety assessment. Two safety endpoints and one tolerability endpoint were assessed at these visits and used to inform the adaptive design, namely: (1) the development of a resting tachycardia, defined as any heart rate greater than 120 bpm or greater than 110 bpm on two consecutive assessments 15 minutes apart, measured at time points estimated to correspond to maximal serum drug concentration, occurring either 14 or 28 days after treatment initiation, with the highest acceptable proportion of patients experiencing this safety endpoint set at 20%; (2) the development of ECG changes suggestive of ischemia on maximal treadmill testing at the 28 day visit, with a maximum tolerable fraction of patients experiencing this event set at 20%; and (3) the discontinuation of the study medication, for any reason, with the maximum tolerable limit on this safety endpoint set at 40%. The first two endpoints, tachycardia and ischemia, were considered safety endpoints while the discontinuation of study medication was considered the tolerability endpoint.

Study subjects were evaluated for the occurrence of multiple hemodynamic abnormalities at two weeks and for resting tachycardia or ECG changes suggestive of ischemia at four weeks. While the four-week evaluation was considered the primary safety evaluation, any subject who experienced resting tachycardia or other hemodynamic changes requiring withdrawal of the study medication at two weeks would be included in the group of subjects who had discontinued the medication when evaluating the tolerability of the study medications. Further, if the specific reason the study medication was discontinued at two weeks was resting tachycardia, then the patient was also considered to have met the criterion for the resting tachycardia adverse effect at four weeks. Thus, at the time of the interim analysis, it was anticipated that there would be patients whose data from the two-week visit were known but whose four-week data were not yet available. This approach was taken to most rapidly identify those patients experiencing adverse effects of the investigational medication.

### Definition of the Adaptive Design

The first adaptive interim analysis was planned to occur after 4-week visit data were available from 50 patients, at which time data from approximately 10 subjects in each of the K-134-containing arms would be available. If deemed necessary by the independent data monitoring committee (DMC), the protocol specified that a second adaptive interim analysis could be conducted after 4-week visit data were available from 100 patients, at which time approximately data from 20 subjects in each of the K-134-containing arms would be available.

**Figure 1:**
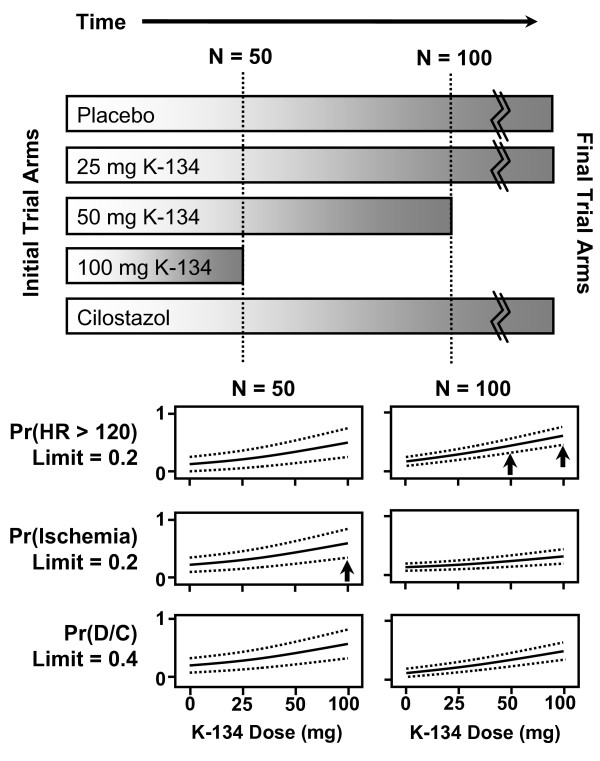
**Schematic of Adaptive Arm-dropping Strategy**. This figure illustrates the adaptive arm-dropping strategy planned for the trial, based on the use of logistic dose-response models to integrate information from the placebo and all K-134 containing arms. Initially, the trial begins with equal randomization to the placebo, cilostazol, and the three K-134-containing arms. At each of two planned interim analyses, occurring when 28-day data are available from either 50 or 100 patients, logistic dose-response models are fit for each of the three safety and tolerability endpoints (resting tachycardia [denoted HR > 120], signs of ischemia on ECG, and medication discontinuation), using data from the placebo and K-134-contining arms. If the lower limit of the 80% confidence interval around the logistic model (the upper and lower limits are illustrated by dotted lines) exceeds the maximum tolerable limit of the safety or tolerability endpoint at a particular dose (excluding placebo), then that arm of the trial is to be discontinued. In the hypothetical scenario shown in the figure, the ischemia safety endpoint limit is exceeded (arrow) when N = 50 for the highest (100 mg) dose of K-134 so that arm is discontinued and the four remaining arms are continued until N = 100. At the second interim analysis, however, the maximum tolerable rate for resting tachycardia is exceeded by the lower limit of the 80% confidence interval at both the 50-mg and 100-mg doses (arrows). Since the 100-mg dose was already discontinued in this hypothetical example at the 50-patient review, the 50-mg dose arm would be discontinued at this point and new research subjects would be randomized in a balanced manner to one of the three remaining arms for the remaining duration of the trial.

For each adaptive interim analysis, dose-response models for each of the three safety and tolerability endpoints (resting tachycardia, ECG changes suggestive of ischemia, and discontinuation of study medication) were developed. The guideline for dropping of a dose was based on the 80%, two-tailed confidence interval for the incidence of each endpoint at each dose, based on these logistic dose-response models. The use of dose-response modeling allowed information from all doses, including placebo, to contribute to the estimates obtained at each dose. If the 80% confidence interval for the fraction of patients experiencing the safety or tolerability endpoint lied completely above the maximum tolerable limit for a given dose, then that dose and any higher doses were to be discontinued (Figure 1).

The use of an 80% confidence interval for this purpose was based on Monte Carlo simulations of thousands of repetitions of the proposed clinical trial, using a variety of coverage probabilities, and under a variety of assumptions regarding true rates of adverse events (either the adverse effects of resting tachycardia, ECG changes suggesting ischemia, or patients discontinuing their study medication) with the different doses of K-134. Thus, the 80% coverage probability was not selected based on a theoretical consideration but, instead, based on the observation that this approach provided reasonable assurance of retaining treatment arms with acceptable adverse event rates while, at the same time, provided a high probability of dropping an arm that was unsafe or poorly tolerated. It is useful to note that the 80% coverage probability is more aggressive in allowing the termination of arms with potential safety or tolerability problems--it results in our requiring less evidence to drop an arm compared to what would have happened if we had used 95% confidence intervals.

The overall goal was to retain the two highest-dose arms with acceptable safety and tolerability event rates, assuming two such arms existed. However, if both the 50-mg and 100-mg arms were found to have unacceptable dose-limiting event rates, the trial was to be continued with only a single K-134-containing arm (the 25-mg arm). If all three K-134-containing arms were found to have unacceptable dose-limiting event rates, the DMC was to recommend early termination of the trial. While this guideline for the dropping of trial arms was predefined, the DMC was empowered to use judgment and all available information in implementing the dose arm stopping guidelines, for example, if some of the information was internally inconsistent or additional information (e.g., the event rate in the cilostazol arm or unanticipated adverse events) was deemed relevant.

## Results

### Meetings of the Independent Data Monitoring Committee (DMC)

A first, inaugural meeting of the DMC was held on July 1, 2008 and included representatives of the sponsor, Steering Committee, project support personnel, and members of the DMC. Background information regarding preclinical and prior clinical experience with K-134 was provided and the rationale for using an adaptive dose ranging approach was discussed. At this meeting, the operating characteristics of the planned approach to treatment arm selection was presented by the statistician member of the DMC, based on simulation studies conducted by that DMC member (see Additional File 1), and unanimously judged to be acceptably accurate in both the dropping of arms with unacceptable safety or tolerability profiles and in retaining arms with acceptable characteristics. In addition, to discussing logistical issues, the group reviewed the draft DMC Charter (see Additional File 2) and approved the adaptive dose finding strategy as presented.

During the second DMC meeting on August 26, 2008, the DMC reviewed and approved the trial protocol. The first subject was randomized on December 5, 2008. The DMC met for a third time on April 21, 2009 by teleconference to conduct a "dry run" for the upcoming interim analysis meeting, during which a decision regarding dropping one or more treatment arms would be considered. During this meeting, issues regarding the recording, definition, and tabulation of key endpoints were discussed. For example, clarifying that for subjects who had completed the 14-day, but not the 28-day visit, the heart rate measured on day 14 alone could be used to define the safety endpoint of resting tachycardia. Although by prior agreement no consideration of termination of a treatment arm was considered at this meeting, the group reviewed current logistic dose-response modeling results to ensure the analysis approach was well understood by the group. At that time, data from anywhere between 11 and 21 subjects were available for each of the three safety and tolerability endpoints (distributed across all five arms).

In prior meetings, the DMC had been informed that the sponsor was simultaneously conducting a trial of K-134 in Japan. While it was acknowledged by all that the patient populations and pharmacokinetics would likely be different in the Japanese versus the American and Russian populations, the DMC felt that adverse event data from the Japanese trial might be helpful in interpreting the sparse safety data expected to be available early in the current trial. Based on the rationale that the DMC should be provided with access to safety and tolerability information from all reliable sources, the DMC requested serious adverse event and treatment assignment information from the ongoing Japanese trial. The sponsor agreed to this request and provided serious adverse event and sealed treatment assignment information (with language translation) to the DMC chair prior to the adaptive interim analysis meeting. No sponsor personnel were unblinded to treatment assignment.

### Interim Analysis Results and Adaptive Decision Making

At the fourth meeting of the DMC, held on July 10, 2009, a total of 199 subjects had been randomized and 28-day visit data were available from 143. A rapidly-increasing rate of subject enrollment and scheduling constraints resulted in data from a larger sample being available at the time of the first interim analysis than originally planned. Thus, at the time of this interim analysis, 28-day visit data were available from a larger number of subjects than had been planned, even for the second interim analysis. In open session, the DMC Chair reviewed the statistical guidelines for the planned arm-dropping adaptation and informed the group that the serious adverse event data from the concurrent Japanese trial were available for closed review by the DMC if deemed necessary. In closed session, the DMC discussed details of the closed reports, focusing on data quality in the reporting of the corrected QT interval (QTc) obtained from ECGs, and were informed that the statistical programs used to create the adaptive endpoint dose-response analyses had been independently validated by the unblinded statistician assigned to the DMC. The Committee also reviewed all adverse event data from the study and identified no substantial concerns. Based on this observation, the Committee voted unanimously not to review the sealed Japanese serious adverse event data that had been provided, based on knowledge of the total number of serious adverse events that had occurred in the Japanese trial and the rationale that no reasonably plausible pattern in serious adverse events in the Japanese trial would warrant an alteration in the current trial.

The observed dose-response models for each of the three safety and tolerability endpoints were then considered (Table 1). No subject in any of the five treatment arms (placebo, three K-134-containing arms, and cilostazol) demonstrated resting tachycardia or ischemic changes on ECG meeting the pre-specified criteria. As mentioned above, the number of subjects available was greater than the originally-planned number. For example, a total of 115 subjects were included in the dose-response model for tachycardia, with 85 receiving K-134, although some of these had only 2-week data available. However, due to limitations in data availability, only 63 subjects were included in the model for ischemic changes, with 43 receiving K-134. Three of the 25 (12%) subjects receiving 100 mg of K-134 had discontinued their medication; 1 of 32 (3%) subjects receiving 50 mg and 1 of 28 subjects (4%) receiving 25 mg had discontinued. No subjects (0 of 32 [0%]) receiving placebo had discontinued. Since the observed discontinuation rate (12%) at the highest dose was still well below the maximum allowed rate of 40%, there was no suggestion of unanticipated tolerability concerns in any of the K-134-containing arms. Based on the lack of any concerning safety or tolerability endpoint data, the DMC recommended the termination of the 25-mg arm, applying the predefined strategy of retaining the two highest safe and tolerable K-134-containing arms. This recommendation was presented to and accepted by the trial's Steering Committee immediately following the closed DMC meeting and subsequently implemented by the sponsor.

**Table 1:** Safety and Tolerability Endpoints at Time of Interim Analysis on July 10, 2009*

Endpoint	Trial Arm	Observed Rate	Model-based Rate (80% CI)
	Placebo	0 of 30 (0%)	0.0 (---, ---)
	
	25 mg K-134	0 of 29 (0%)	0.0 (---, ---)
	
Resting Tachycardia	50 mg K-134	0 of 30 (0%)	0.0 (---, ---)
	
	100 mg K-134	0 of 26 (0%)	0.0 (---, ---)
	
	Cilostazol	0 of 28 (0%)	

	Placebo	0 of 20 (0%)	0.0 (---, ---)
	
	25 mg K-134	0 of 14 (0%)	0.0 (---, ---)
	
Ischemic ECG Changes	50 mg K-134	0 of 19 (0%)	0.0 (---, ---)
	
	100 mg K-134	0 of 10 (0%)	0.0 (---, ---)
	
	Cilostazol	0 of 15 (0%)	

	Placebo	0 of 32 (0%)	0.009 (0.002, 0.038)
	
	25 mg K-134	1 of 28 (4%)	0.018 (0.004, 0.072)
	
Discontinuation	50 mg K-134	1 of 32 (3%)	0.035 (0.009, 0.13)
	
	100 mg K-134	3 of 25 (12%)	0.12 (0.032, 0.37)
	
	Cilostazol	1 of 27 (0%)	

*See text for definitions of safety and tolerability endpoints.

### Implementation of the Adaptation

To implement the adaptive elimination of the 25-mg dose arm, the sponsor and contractor supporting the trial rapidly took steps: (1) to modify the trial's randomization system so no new subjects would be assigned to the 25-mg arm; (2) to obtain a listing of all subjects previously randomized to the 25-mg arm while not compromising the blinding of other subjects; (3) to notify clinical study sites which of their patients should be scheduled for study termination; (4) to communicate to each site that this termination was based on the pre-specified adaptive design but not to identify which treatment arm was to be discontinued; and (5) to revise the total planned sample size for the trial, allowing for the subjects being discontinued because of prior allocation to the 25-mg K-134 arm. These steps were completed within 3 days of the July 10 meeting. The communication sent to each clinical trial investigator emphasized that the discontinuation of subjects was part of the prespecified adaptive design of the study and that the communication should be shared with the Institutional Review Board (IRB) or Institutional Ethics Committee (IEC) at their institution. Information regarding which arm was being discontinued was known only to the Steering Committee and key personnel at the sponsor, and was not communicated to site investigators. Lastly, the subjects' study physicians were instructed to conduct a final evaluation and the patients were transferred to standard care according to their personal physicians' preferences.

In follow up to the July 10, 2009 meeting of the DMC, the sponsor identified a number of factors that contributed to the missing data at the time of review regarding ST segment depression and related ECG outcomes at day 28. The availability of data elements at the time of the formal interim analysis was influenced by multiple factors, including: (1) the timing of data collection for each element; (2) the time delay associated with central laboratory ECG reading; and (3) time required for the submission of CRFs and the monitoring of data from each site. The larger quantity of missing ECG data was largely the result of the time required for receipt and central over-reading of ECGs.

A repeat analysis of the three safety and tolerability endpoints with a more complete data set was conducted on September 23, 2009 demonstrating that none of 130 (0%) subjects receiving K-134 demonstrated tachycardia at either 14- or 28-days of treatment and none of 96 subjects (0%) receiving K-134 had exhibited ischemic ECG changes at 28 days (Table 2). The overall discontinuation rates were verified to be consistent with those seen in July. Although the DMC's adaptation recommendation had been provided, accepted, and implemented, the DMC continued to receive and review safety and tolerability endpoint dose-response models periodically.

**Table 2:** Safety and Tolerability Endpoints as of September 23, 2009*

Endpoint	Trial Arm	Observed Rate	Model-based Rate (80% CI)
	Placebo	0 of 57 (0%)	0.0 (---, ---)
	
	25 mg K-134	0 of 37 (0%)	0.0 (---, ---)
	
Resting Tachycardia	50 mg K-134	0 of 52 (0%)	0.0 (---, ---)
	
	100 mg K-134	0 of 50 (0%)	0.0 (---, ---)
	
	Cilostazol	0 of 53 (Z%)	

	Placebo	0 of 43 (0%)	0.0 (---, ---)
	
	25 mg K-134	0 of 29 (0%)	0.0 (---, ---)
	
Ischemic ECG Changes	50 mg K-134	0 of 45 (0%)	0.0 (---, ---)
	
	100 mg K-134	0 of 42 (0%)	0.0 (---, ---)
	
	Cilostazol	0 of 44 (Z%)	

	Placebo	2 of 63 (3%)	0.021 (0.008, 0.050)
	
	25 mg K-134	41 of 42 (98%)	**
	
Discontinuation	50 mg K-134	2 of 61 (3%)	0.055 (0.023, 0.13)
	
	100 mg K-134	9 of 60 (15%)	0.14 (0.061, 0.28)
	Cilostazol	5 of 57 (9%)	

*See text for definitions of safety and tolerability endpoints.

**Nearly all patients discontinued due to arm dropping at previous analysis;

25 mg dose not included in logistic regression.

On November 24, 2009, the DMC met a fifth time to conduct a detailed review of serious adverse events (SAEs) in the trial. This review was conducted in an unblinded manner in closed session. It was noted that a substantial fraction of all SAEs (35% or 6 of 17) had occurred in the placebo arm. Further, there was no apparent difference in the incidence of SAEs by country or by subject age.

In consultation with the trial's Steering Committee, the DMC agreed that further DMC oversight should be focused on the review of SAE and adverse event (AE) experience in the trial. The DMC met a sixth time on February 3, 2010 to review both AE and SAE information. The DMC noted potential dose-response relationships for K-134 with the occurrence of palpitations, gastrointestinal disorder (e.g., diarrhea, nausea) and edema. Tachycardia and hypovolemia were specifically noted to be absent from AE reports. At the DMC meetings of July 10, 2009, November 24, 2009, and February 3, 2010, the DMC reviewed detailed tabulations and graphical presentations of hemodynamic and ECG data. No concerning patterns were observed, although the changes expected in the cilostazol arm were noted. The study was successfully completed with last patient visit occurring July 7, 2010 and database lock occurring July 28, 2010. All contents of this manuscript were embargoed within the DMC until after database lock had been confirmed.

## Discussion

The clinical trial described here represents the first application of prospectively defined, adaptive decision-making in a PAD trial. By addressing pre-trial uncertainty in the maximum safe and well-tolerated dose, and the goal of obtaining preliminary efficacy data from the maximum tolerated dose, this approach allowed the initial evaluation of a broad range of doses, followed by the focusing of trial resources and human subjects on the most promising treatment arms. The selection of treatment arms to carry forward was based on safety and tolerability endpoints, rather than the primary efficacy outcome, reflecting the expectation that dose-limiting side effects would appear quickly whereas evidence of efficacy might not appear until the completion of treatment.

If, instead of dropping the 25-mg K-134 treatment arm, all arms had been continued to full enrollment, then approximately 43 additional research subjects would have been required to complete the trial (that is a minimum of 85 subjects in all arms). This additional enrollment would have increased the cost and duration of the trial, and resulted in the exposure of additional subjects to K-134, but without providing relevant additional information on either the safety or efficacy of K-134 at doses likely to be clinically active.

Even when prospectively defined, adaptive clinical trials are inherently more complex than traditional, parallel-armed designs because of the multiple ways the clinical trial may "play out" when implemented. Thus, it is often difficult or impossible to directly calculate the power, type I error rate, or the expected accuracy in dose selection for a particular trial design. Simulations of the proposed trial design are often required to verify acceptable operating and performance characteristics. In practice, however, a general approach is to use simulations to define the characteristics of a proposed design, to adjust design parameters (e.g., thresholds for adding or dropping arms, adjusting randomization proportions, etc.) based on the simulation results, and then to repeat the simulations. This iterative process is repeated until the appropriate balance of trial size, power, and type I error risk is obtained. In a learn-phase trial such as this one, defining this appropriate balance is largely a subjective process. When an adaptive design is used in the setting of a confirmatory, phase III trial, the required numerical simulations are often quite extensive because of the importance of precisely defining the trial's operating characteristics under a wide range of null and alternative hypotheses. In the current setting, however, the trial results were to be used to inform internal decision making by the sponsor and, accordingly, the numerical simulations were more limited.

The complexity of the adaptive design is also reflected in the trial's actual execution. While the original intent was to conduct a formal assessment of the adaptive design criteria after data from 50 patients had been accrued through four weeks of treatment, in actuality the review took place after many more patients had been enrolled. This reflected an unanticipated acceleration of enrollment, inherent delays in entering data into and verifying data in the database, and the challenges of scheduling meetings. Future trials can mitigate the risk of delay by taking steps to better coordinate the start-up of new sites and minimizing data entry delays. If serious safety concerns exist, enrollment can be suspended pending the adaptive decision making review. This step was considered and felt unnecessary in the current trial. Safety risks can be further minimized by ensuring ongoing, unblinded review of all serious adverse events as was done in the current trial.

The development of the adaptive design significantly accelerated the clinical development of K-134. The Steering Committee was faced with requiring a phase I study in patients with PAD to define the safety and tolerability of K-134 and thus delaying development by 9-12 months, excluding the 100 mg arm from the phase II trial and thus risk excluding the optimal dose, or conducting a large phase II trial fully populating all five study arms. The latter option would have resulted in increased cost and time due to the additional enrollment requirements as noted above. In practice the integration of safety-specific assessments at early time points, the use of the adaptive design, and an empowered and experienced independent DMC allowed the trial to be completed and subject safety optimized.

In addition to ultimately achieving efficiency in the allocation of resources, the process of designing a prospectively-defined adaptive trial requires and facilitates a careful consideration of trial goals and the adequacy of pre-trial information regarding the likely maximum tolerable dose, the effective dose range, and the treatment effect. Similarly, the process of planning for an adaptive interim analysis, which necessarily includes considerations of data quality, availability, and other logistical issues, may lead to better planning for the logistical support and conduct of the trial itself.

## Conclusions

In this phase II, dose-finding trial of K-134 in the treatment of stable intermittent claudication, no concerning safety or tolerability signals were seen at interim analysis, allowing the discontinuation of the lowest-dose-containing arm and the retention of the two highest-dose-containing arms.

## Abbreviations

ABI: Ankle Brachial Index; AE: Adverse Event; bpm: Beats per minute; CI: Confidence Interval; CPC: Colorado Prevention Center; CRF: Case Report Form; DMC: Data Monitoring Committee; ECG: Electrocardiogram; HR: Heart Rate; IA: Interim Analysis; IEC: Institutional Ethics Committee; IRB: Institutional Review Board; IVRS: Interactive Voice Recognition System; KRI: Kowa Research Institute; mg: milligram; PAD: Peripheral Artery Disease; PDE: Phosphodiesterase; QTc: Corrected QT Interval; SAE: Serious Adverse Event; WIRB: Western IRB

## Competing interests

Members of the Data Monitoring Committee (DMC) and the Steering Committee received compensation from the sponsor for the time and effort devoted to the conduct and oversight of the trial. However, this compensation was in no way related to the outcome of the trial, nor do any of the authors have any financial interest in the sponsor or pharmacologic agent being evaluated. This information regarding conflicts of interest is included in the manuscript as requested in your Instructions for Authors. Also note that Dr. Hiatt is President of CPC Clinical Research, a non-profit academic research organization responsible for trial management.

## Authors' contributions

RJL chaired the DMC and drafted the manuscript. JTC served as a member of the DMC, conducted clinical trials simulations, performed the modeling required to implement the adaptive algorithm, and drafted the Appendix. JRT and JRM served as members of DMC. EPB served as the chair of the trial's Steering Committee and LTC and WRH served as members. All authors participated in the planning and oversight of the clinical trial as well as in the review and revision of the manuscript. All authors read and approved the final manuscript.

## Supplementary Material

Additional file 1 Appendix 1 - Operating Characteristics of Adaptive Design as Determined by Simulation Click here for file

Additional file 2 Appendix 2 - Data Monitoring Committee Charter Click here for file
