# Supplementary material for: Application of adaptive design and decision making to a phase II trial of a phosphodiesterase inhibitor for the treatment of intermittent claudication
Source: Trials. 2011 May 25;12:134. doi: 10.1186/1745-6215-12-134 (PMC3126735; doi:10.1186/1745-6215-12-134)
Supplement: Additional file 1 — Appendix 1 - Operating Characteristics of Adaptive Design as Determined by Simulation [file 1745-6215-12-134-S1.DOC]

**Appendix 1 – Operating Characteristics of Adaptive Design as Determined by Simulation**

**Operating Characteristics:** We calculated the design’s operating characteristics via simulation. We simulated 1,000 trials for each of 8 scenarios. Simulations assumed the adverse event (AE) rates shown in each of the following tables and the probability of each AE was assumed to be independent of having another type of adverse event.

Table 1 (below) shows the assumed true event rates and operating characteristics for scenario 1.

| *Scenario 1*  True AE rate | Dose | | | |
| --- | --- | --- | --- | --- |
|  | 0 mg | 25 mg | 50 mg | 100 mg |
| Tachycardia | 5% | 5% | 5% | 5% |
| ECG Changes | 5% | 5% | 5% | 5% |
| Discontinuation | 10% | 10% | 10% | 10% |
| Fraction Dropped at IA 1 | 0% | 0% | 0% | 0% |
| Fraction Dropped at IA 2 | 0% | 0% | 0% | 0% |

Under these conditions, no doses were ever dropped at either interim analysis (IA) in 1000 simulations. This was reassuring as it suggested that the risk of inappropriately discontinuing an arm based on the low frequency event rates was very low.

Table 2 shows the assumed true event rates and operating characteristics for scenario 2.

| *Scenario 2*  True AE rate | Dose | | | |
| --- | --- | --- | --- | --- |
|  | 0 mg | 25 mg | 50 mg | 100 mg |
| Tachycardia | 5% | 10% | 10% | 10% |
| ECG Changes | 5% | 10% | 10% | 10% |
| Discontinuation | 10% | 20% | 20% | 20% |
| Fraction Dropped at IA 1 | 0% | 0% | 0% | 3% |
| Fraction Dropped at IA 2 | 0% | 0% | 0% | 3% |

The highest dose was dropped in 3% of the simulated trials as shown.

Table 3 shows the assumed true event rates and operating characteristics for scenario 3.

| *Scenario 3*  True AE rate | Dose | | | |
| --- | --- | --- | --- | --- |
|  | 0 mg | 25 mg | 50 mg | 100 mg |
| Tachycardia | 5% | 15% | 15% | 15% |
| ECG Changes | 5% | 15% | 15% | 15% |
| Discontinuation | 10% | 20% | 30% | 30% |
| Fraction Dropped at IA 1 | 0% | 0% | 1% | 18% |
| Fraction Dropped at IA 2 | 0% | 0% | 1% | 22% |

The highest dose was dropped 18% of the time at the first interim analysis and an additional 4% of the time at the second interim analysis. The 50 mg dose was dropped 1% of the time.

Table 4 shows the assumed true event rates and operating characteristics for scenario 4.

| *Scenario 4*  True AE rate | Dose | | | |
| --- | --- | --- | --- | --- |
|  | 0 mg | 25 mg | 50 mg | 100 mg |
| Tachycardia | 5% | 15% | 15% | 20% |
| ECG Changes | 5% | 15% | 15% | 15% |
| Discontinuation | 10% | 20% | 30% | 30% |
| Fraction Dropped at IA 1 | 0% | 0% | 1% | 24% |
| Fraction Dropped at IA 2 | 0% | 0% | 2% | 33% |

The highest dose was dropped 24% of the time at the first interim analysis and an additional 9% of the time at the second interim analysis. The 50 mg dose was dropped 2% of the time.

Table 5 shows the assumed true event rates and operating characteristics for scenario 5.

| *Scenario 5*  True AE rate | Dose | | | |
| --- | --- | --- | --- | --- |
|  | 0 mg | 25 mg | 50 mg | 100 mg |
| Tachycardia | 5% | 15% | 15% | 20% |
| ECG Changes | 5% | 15% | 15% | 15% |
| Discontinuation | 10% | 20% | 30% | 40% |
| Fraction Dropped at IA 1 | 0% | 0% | 1% | 31% |
| Fraction Dropped at IA 2 | 0% | 0% | 1% | 43% |

The highest dose was dropped 31% of the time at the first interim analysis and an additional 12% of the time at the second interim analysis. The 50 mg dose was dropped 1% of the time. These results emphasize the liberal nature of the guidelines, as even high event rates for the outcomes of interest are tolerated with high frequency.

Table 6 shows the assumed true event rates and operating characteristics for scenario 6.

| *Scenario 6*  True AE rate | Dose | | | |
| --- | --- | --- | --- | --- |
|  | 0 mg | 25 mg | 50 mg | 100 mg |
| Tachycardia | 5% | 15% | 20% | 20% |
| ECG Changes | 5% | 15% | 15% | 15% |
| Discontinuation | 10% | 25% | 40% | 40% |
| Fraction Dropped at IA 1 | 0% | 0% | 3% | 40% |
| Fraction Dropped at IA 2 | 0% | 0% | 4% | 55% |

The highest dose was dropped 40% of the time at the first interim analysis and an additional 15% of the time at the second interim analysis. The 50 mg dose was dropped 4% of the time. A comparison of Tables 5 and 6 illustrates the utility of using data from all dosing groups in an integrated fashion, as the outcome of the 50 mg arm appropriately influences the decision regarding the 100 mg arm.

Table 7 shows the assumed true event rates and operating characteristics for scenario 7.

| *Scenario 7*  True AE rate | Dose | | | |
| --- | --- | --- | --- | --- |
|  | 0 mg | 25 mg | 50 mg | 100 mg |
| Tachycardia | 5% | 15% | 20% | 25% |
| ECG Changes | 5% | 15% | 20% | 25% |
| Discontinuation | 5% | 15% | 15% | 20% |
| Fraction Dropped at IA 1 | 0% | 0% | 3% | 49% |
| Fraction Dropped at IA 2 | 0% | 0% | 5% | 67% |

The highest dose was dropped 49% of the time at the first interim analysis and an additional 18% of the time at the second interim analysis. The 50 mg dose was dropped 5% of the time.

Table 8 shows the assumed true event rates and operating characteristics for scenario 8.

| *Scenario 8*  True AE rate | Dose | | | |
| --- | --- | --- | --- | --- |
|  | 0 mg | 25 mg | 50 mg | 100 mg |
| Tachycardia | 5% | 15% | 20% | 30% |
| ECG Changes | 5% | 15% | 20% | 25% |
| Discontinuation | 10% | 25% | 40% | 50% |
| Fraction Dropped at IA 1 | 0% | 1% | 6% | 72% |
| Fraction Dropped at IA 2 | 0% | 1% | 10% | 90% |

The highest dose was dropped 72% of the time at the first interim analysis and an additional 18% of the time at the second interim analysis. The 50 mg dose was dropped 10% of the time.
