# Supplementary material for: Application of adaptive design and decision making to a phase II trial of a phosphodiesterase inhibitor for the treatment of intermittent claudication
Source: Trials. 2011 May 25;12:134. doi: 10.1186/1745-6215-12-134 (PMC3126735; doi:10.1186/1745-6215-12-134)
Supplement: Additional file 2 — Appendix 2 - Data Monitoring Committee Charter [file 1745-6215-12-134-S2.DOC]

**Appendix 2 – Data Monitoring Committee Charter**

**1.0 Introduction**

This Charter is for the Data Monitoring Committee (DMC) for a phase II, multicenter clinical trial of K-134 in the treatment of intermittent claudication. The trial is entitled “A Phase IIa Multicenter, Randomized, Double-blind, Placebo- and Active-controlled, Parallel-Group Study to Evaluate the Safety and Efficacy of K-134 for the Treatment of Intermittent Claudication.” The trial is supported by Kowa Research Institute, Inc. (KRI) through a contract with the Colorado Prevention Center (CPC).

This Charter is a living document that may be revised by the DMC as required to facilitate their role in providing trial guidance, oversight, and protecting human subjects from avoidable risk. Such revisions will be subject to approval of the DMC, the trial’s Steering Committee, and KRI.

1. **Role of the Data Monitoring Committee (DMC)**
   1. General Role and Responsibilities

The role of the DMC is to act in an independent, advisory capacity to the trial’s Steering Committee, providing guidance to help ensure:

- The protection of human subjects participating in the study;
- The proper conduct of the planned adaptive trial design and interpretation of interim analyses; and
- The ongoing scientific validity, integrity, and clinical and scientific relevance of the study.

The DMC will provide recommendations about starting, continuing, modifying, and/or stopping the study based on considerations of patient safety and trial futility as appropriate. In addition, the DMC may make recommendations to the trial’s Steering Committee about:

- Definitions of and responses to adverse events and patterns in adverse events;
- Value of procedures and participant burden;
- Selection, recruitment, and retention of participants;
- Adherence to protocol requirements;
- Completeness, quality, and analysis of measurements;
- Amendments to the study protocol and consent forms;
- Performance of individual centers and core labs; and
- Participant safety.

Please see Section 4.4 for information regarding the communication of recommendations from the DMC.

- 1. Role of DMC in Initiation of Patient Enrollment

Prior to the initiation of patient enrollment, the DMC must review the study protocol and forward their recommendations, if any, to the chair of the Steering Committee. Any substantive changes to the approved clinical protocol must be reviewed by the DMC.

- 1. Role of DMC in Reviewing and Monitoring Adverse Events (AEs)

Adverse event data, including information regarding adverse events (AEs) and serious adverse events (SAEs), will be reported by investigators to the sponsor or their designee, as detailed in the protocol. SAE reports and follow up information related to prior reports will be forwarded to the DMC chair for review every 2 weeks. Tabulations of AEs and SAEs will be reviewed by the DMC at each meeting which occurs after the initiation of patient enrollment.

- 1. Role of DMC in Interim Safety Analyses

At the time of each interim analysis, the DMC will review data related to study progress and safety of the study arms. The DMC deliberations will be guided by the trial design with adaptive dose selection, as defined in the study protocol and the statistical analysis plan, although the DMC may make recommendations that deviate from the approved trial design if necessary to protect patient safety, or based on considerations of harm or trial futility. DMC recommendations for the continuation, termination, or modification of the trial will be communicated to the trial’s Steering Committee, as detailed in Section 4.4.

**3.0 Membership**

The DMC will consist of a minimum of four members (including both voting and non-voting members) with, collectively, expertise in clinical research methodology and the treatment of cardiovascular disease. An unblinded statistician, whose role will include the production of data tables and reports for DMC review, will serve as a non-voting member of the DMC. The initial membership is given in Appendix 1. In the event that a DMC member is unable to complete their duties, a suitable replacement will be identified by the trial’s Steering Committee and/or DMC Chair, subject to approval by a majority of the remaining DMC members *and* the trial’s Steering Committee.

All DMC members must be free of substantive conflicts of interest, including financial, scientific, or personal conflicts of interest, with respect to the clinical study, the principal and coinvestigators, the trial’s Steering Committee, KRI, and CPC. All potentially-relevant conflicts of interest must be disclosed by potential DMC members prior to appointment. Whether a potential conflict of interest is, in fact, substantive and disqualifying will be determined by consensus of the trial’s Steering Committee, the DMC chair, and the CPC.

A quorum will require two voting DMC members in addition to the chair. In an extraordinary circumstance in which the chair is unable to participate in DMC deliberations, and an urgent DMC meeting is required to ensure research subject safety, an acting DMC chair may be selected by the DMC members, from the pool of existing DMC members.

1. **DMC Meetings**
   1. Calling of Meetings

A meeting of the DMC may be called at any time by the chair of the DMC, the trial’s Steering Committee, or the CPC. If the DMC chair and trial’s Steering Committee or the CPC are in disagreement regarding the need for a DMC meeting, the opinion of the DMC chair will prevail.

The purpose of the first meeting will be to review and discuss this Charter, to provide an overview of the K-134 development program and prior clinical experience, to review and make recommendations about the proposed protocol, and to determine the frequency of DMC meetings. In addition, the details of the planned dose finding adaptation strategy will be discussed.

Subsequent DMC meetings will be held when the results of each planned interim analysis become available (this includes the planned adaptation points), and not less frequently than annually. DMC meetings may be called at any time in response to adverse events or other subject experience. Additional meetings may be called as above.

- 1. Meeting Formats

DMC meetings may be held in person, by telephone conference, or a combination of the two. DMC sessions may be either *open* or *closed* and, in general, each DMC meeting will include both an open and a closed session. During an open session, the principal investigator or coinvestigators, representatives of the trial’s Steering Committee, employees of KRI, representatives of the CPC and its delegates, and/or other interested parties may be present. During open sessions, only non-confidential information that does not threaten the integrity or feasibility of the study will be discussed, such as general information regarding patient enrollment, aggregate outcome and safety data, amendments and modifications to the protocol, and external information which may impact the conduct of the study.

Closed DMC sessions may only include full voting members of the DMC, the unblinded statistician/non-voting member responsible for creation of reports to be reviewed by the DMC in closed sessions, and personnel whose presence is explicitly determined to be required by a majority vote of the DMC. All matters and information, including unblinded data, impacting the safety, ethics, and scientific validity and integrity of the study may be discussed during closed sessions. All formal recommendations considered by the DMC will be discussed during closed sessions.

Voting on recommendations will follow Roberts’ Rules of Order (Robert's Rules of Order Newly Revised (10th Edition) by Henry M. Robert III, William J. Evans (Editor), Daniel H. Honemann (Editor), Thomas J. Balch (Editor), Sarah Corbin Robert, Henry M. Robert III, General Henry M. Robert).

At the conclusion of the closed session, participants in the open session may be re-convened so that the DMC chair may provide a summary of the DMC’s recommendations, if applicable, and to provide an opportunity for members of the trial’s Steering Committee or representatives of KRI or the CPC to obtain clarification regarding the recommendations.

- 1. Minutes

The minutes of open and closed sessions will be prepared by the DMC chair and the unblinded statistician. The minutes of open sessions may be distributed freely (e.g., to other Steering Committee members, KRI or CPC representatives), as deemed appropriate by the trial’s Steering Committee and DMC chair. The minutes of closed sessions may only be distributed to personnel present at those sessions, until after the formal termination of the study. Once the study is formally terminated and all statistical analyses have been completed, the minutes of closed sessions will be released by the DMC chair.

- 1. DMC Communications and Recommendations

As soon as possible following each DMC meeting, the DMC Chair will communicate the results of the meeting to the chair of the trial’s Steering Committee as follows. DMC communication(s) and/or recommendation(s) will be transmitted in written format to the chair of the trial’s Steering Committee, who will review the information and, as appropriate, forward it to other members of the trial’s Steering Committee, the principal investigator, coinvestigators, and/or representatives of the CPC and KRI. The actual transmission of DMC communications or recommendations to the Steering Committee chair may occur by electronic mail or by facsimile. The rationale for a DMC recommendation may or may not be given, consistent with maintaining the scientific integrity of the study.

If, in the opinion of the DMC, rapid communication of information or recommendations from the DMC to trial investigators is required to ensure the safety of study participants or the integrity of the trial, then the DMC may transmit information or recommendations simultaneously to the Steering Committee chair and trial investigators, but every effort will be made to coordinate communication with the trial’s Steering Committee and CPC to avoid confusion. In no case will KRI, the CPC, the trial’s Steering Committee, or the principal investigator prevent or restrict direct communication from the DMC to study personnel if, in the opinion of the DMC, such communication is required to ensure the safety of study participants or trial integrity.

It is expected that trial investigators will not communicate with DMC members about the study directly, except when making presentations or responding to questions at DMC meetings or during conference calls.

If the DMC does not identify any safety or other protocol-related concerns during a meeting then, as soon as possible after the meeting, the chair of the DMC will prepare a Summary Report for distribution to the clinical centers that will state that:

- A review of safety data, adverse events, and information relating to study performance took place on a given date; and
- The DMC recommended that the study continue without modification of the protocol or informed consent.

If concerns are identified or a modification of the trial is recommended, the Summary Report will be modified appropriately. The Summary Report will be transmitted to the trial Steering Committee who will, in general, forward it to the CPC for distribution to trial investigators. If the Summary Report includes recommendations that affect investigator activities or require action on the part of investigators, the Steering Committee may provide additional guidance or instructions for investigators.

- 1. Data Access, Reports, and Statistical Monitoring Plan

To ensure trial integrity, study investigators and other interested parties will not be allowed access to data containing explicit or implicit treatment arm identifiers which, if present, could allow comparisons of outcomes across treatment arms.

To fulfill its duties, the DMC will require access to both blinded and unblinded trial data. Required reports will include, but not necessarily be limited to, information on study enrollment, subject demographics and comorbidities, therapies administered, adverse events (AEs), serious adverse events (SAEs), and outcomes. Initial report content and formats will be determined by the DMC, in collaboration with the trial’s Steering Committee and the CPC as appropriate. Requests for additional reports will be communicated by the DMC chair directly to the unblinded statistician serving on the DMC. In general, the nature of such requests will not be communicated to the trial’s Steering Committee, investigators, or other interested parties, to avoid biasing or impacting study conduct.

For each meeting, the unblinded statistician will prepare summary reports and tables to facilitate the oversight role of the DMC.

- 1. Contractual Considerations

Members of the DMC, including its chair, will be compensated for their time spent on DMC activities by the CPC. This compensation will be based only on time spent directly involved in DMC activities, such as reviewing reports or other information and participating in DMC meetings and correspondence. The nature or timing of DMC recommendations will not impact DMC member compensation.
